# Supplementary material for: Impact of Neonatal Body (Dis)Proportionality Determined by the Cephalization Index (CI) on Gross Motor Development in Children with Down Syndrome: A Prospective Cohort Study
Source: Children (Basel). 2022 Dec 21;10(1):13. doi: 10.3390/children10010013 (PMC9856915; doi:10.3390/children10010013)
Supplement: Supplementary file 1 [file children-10-00013-s001.zip › Supplementary Table S1_01.12.22.pdf]

Table S1. Reliability and content validity of the modified Munich Functional Developmental Diagnostics (MFDD).

**Gross Motor Skills Milestones**	**Scale Mean If Item Deleted**	**Scale Variance If Item Deleted**	**Corrected Item-Total Correlation**	**Squared Multiple Correlation**	**Cronbach's Alpha If Item Deleted**
Lifts head up 90° with a forearm rest	104.18	630.668	0.492	0.823	0.931
Extended arm support	104.18	624.768	0.573	0.679	0.93
Four point kneeling	104.16	643.156	0.326	0.586	0.933
Rolls both ways	104.18	628.222	0.525	0.747	0.931
Reciprocal creeping	104.18	618.077	0.666	0.821	0.929
Semiflexion of the hips and knees	104.21	652.499	0.207	0.596	0.934
Holds body weight on legs when supported in standing	104.12	630.184	0.486	0.739	0.931
Stands up without support	103.98	608.163	0.764	0.969	0.927
Pulls to stand on furniture	103.98	606.418	0.787	0.965	0.927
Walks sideways along furniture	103.87	607.639	0.752	0.883	0.928
Stands without support	103.89	608.897	0.745	0.903	0.928
Walks independently (Walks alone)	103.86	608.306	0.734	0.925	0.928
Stands up without support (Gets to standing without support)	103.79	613.299	0.658	0.796	0.929
Crouches and picks something up without support	103.8	610.561	0.701	0.872	0.928

Walks up one step at a time with rail holding	103.52	626.036	0.492	0.855	0.931
Kicks a stationary ball	103.36	619.325	0.572	0.74	0.93
Walks down one step at a time with rail holding	103.43	624.686	0.501	0.784	0.931
Stands on one foot without help for 2 seconds	102.62	643.802	0.318	0.702	0.933
Jumps in place	102.62	643.548	0.321	0.746	0.933
Follows a toy with eyes	104.14	637.797	0.399	0.59	0.932
In traction - the head follows the torso	104.14	640.197	0.367	0.601	0.933
Positioned, keeps sitting for at least 5 seconds supporting themselves forward	104.14	625.288	0.571	0.674	0.93
Positioned keep sitting for at least 1 minute	104.16	623.956	0.59	0.783	0.93
Sits down alone	104.14	619.797	0.626	0.707	0.929
Sits alone stably	104.18	625.822	0.558	0.785	0.93
