# Supplementary material for: Impact of Neonatal Body (Dis)Proportionality Determined by the Cephalization Index (CI) on Gross Motor Development in Children with Down Syndrome: A Prospective Cohort Study
Source: Children (Basel). 2022 Dec 21;10(1):13. doi: 10.3390/children10010013 (PMC9856915; doi:10.3390/children10010013)
Supplement: Supplementary file 1 [file children-10-00013-s001.zip › Supplementary Table S2_01.12.22.pdf]

Table S2. Levene's test of homogeneity of Munich Functional Developmental Diagnostics (MFDD) motor scales in children with Down syndrome (DS) who had proportionate cephalization index (CI) and disproportionate CI.

Gross Motor Skills Milestones	Levene Statistic	df1	df2	Sig.
Lifts head up 90°, with a forearm rest	0.064	1	54	0.802
Pulls his chest away with his arms outstretched (Extended arm support)	0.606	1	54	0.440
Rises to his knees and palms (Four point kneeling)	1.908	1	54	0.173
Rolls from the back to the abdomen and vice versa (Rolls both ways)	3.451	1	54	0.069
Crawls on hands and knees (Reciprocal creeping)	6.514	1	54	0.014
Semiflexion of the hips and knees	0.304	1	54	0.584
Takes body weight on legs when supported in standing	2.841	1	54	0.098
Stands with support	0.043	1	54	0.837
Pulls to stand on furniture	0.166	1	54	0.685
Walks sideways along furniture	1.948	1	54	0.169
Stands without support	1.603	1	54	0.211
Walks independently (Walks alone)	0.082	1	54	0.775
Stands up without support (Gets to standing without support)	0.899	1	54	0.347
Crouches (Bends) and picks something up without supporting himself	1.719	1	54	0.195
Walks up one step at a time with rail holding	0.039	1	54	0.845
Kicks a stationary ball	0.004	1	54	0.947
Walks down one step at a time with rail holding	0.097	1	54	0.756
Stands on one foot without help 2 s	1.170	1	54	0.284
Jumps in place	1.144	1	54	0.290
Follows the toy from one corner of the eye to another (Follows the toy with his eyes)	3.064	1	54	0.086
In traction, the head follows the torso	0.992	1	54	0.324
Positioned keep sitting for at least 5 s, supporting itself forward (Sits for at least 5 s, supporting itself forward)	1.403	1	54	0.241
Positioned keep sitting for at least 1 minute	0.552	1	54	0.461
Sits down alone	0.650	1	54	0.423
Sits alone stable	1.376	1	54	0.246
