# Supplementary material for: Impact of Neonatal Body (Dis)Proportionality Determined by the Cephalization Index (CI) on Gross Motor Development in Children with Down Syndrome: A Prospective Cohort Study
Source: Children (Basel). 2022 Dec 21;10(1):13. doi: 10.3390/children10010013 (PMC9856915; doi:10.3390/children10010013)
Supplement: Supplementary file 1 [file children-10-00013-s001.zip › Supplementary Table S3_01.12.22.pdf]

Table S3. Equality of group means in children with Down syndrome (DS) with a proportionate cephalization index (CI) and disproportionate CI.

Milestones	Wilks' Lambda	F	df1	df2	P-value
Lifts head up 90° with a forearm rest	0.922	4.575	1	54	0.037*
Extended arm support	0.908	5.481	1	54	0.023*
Four point kneeling	0.997	0.147	1	54	0.703
Rolls both ways	0.999	0.081	1	54	0.777
Reciprocal creeping	1.000	0.012	1	54	0.915
Semiflexion of the hips and knees	0.981	1.044	1	54	0.311
Holds body weight on legs when supported in standing	0.990	0.538	1	54	0.467
Stands up without support	0.999	0.064	1	54	0.801
Pulls to stand on furniture	1.000	0.007	1	54	0.933
Walks sideways along furniture	0.996	0.201	1	54	0.656
Stands without support	0.998	0.092	1	54	0.762
Walks independently (Walks alone)	0.995	0.282	1	54	0.598
Stands up without support (Gets to standing without support)	1.000	0.004	1	54	0.95
Crouches and picks something up without support	0.993	0.393	1	54	0.533
Walks up one step at a time with rail holding	0.997	0.149	1	54	0.701
Kicks a stationary ball	0.968	1.756	1	54	0.191
Walks down one step at a time with rail holding	1.000	0.020	1	54	0.889

Stands on one foot without help for 2 seconds	0.989	0.573	1	54	0.452
Jumps in place	0.989	0.573	1	54	0.452
Follows a toy with eyes	0.945	3.163	1	54	0.081
In traction - the head follows the torso	0.965	1.948	1	54	0.168
Positioned, keeps sitting for at least 5 seconds supporting themselves forward	0.933	3.900	1	54	0.053*
Positioned keep sitting for at least 1 minute	0.939	3.489	1	54	0.067
Sits down alone	1.000	0.030	1	54	0.956
Sits alone stably	1.000	0.050	1	54	0.945

*p<0.05
