# Supplementary material for: Impact of Neonatal Body (Dis)Proportionality Determined by the Cephalization Index (CI) on Gross Motor Development in Children with Down Syndrome: A Prospective Cohort Study
Source: Children (Basel). 2022 Dec 21;10(1):13. doi: 10.3390/children10010013 (PMC9856915; doi:10.3390/children10010013)
Supplement: Supplementary file 1 [file children-10-00013-s001.zip › Supplementary Table S4_01.12.22.pdf]

Table S4. Discriminant analysis of the division of children with Down syndrome (DS) into two groups based on the proportionality of their anthropological measures as disproportionate cephalization index (CI)≥1.1 or proportionate (CI<1.1).

DA Clasification Results[1]			
Category	Predicted Group Membership		Total
	CI<1.1	CI≥1.1	
Original Count CI<1.1	26	4	30
CI≥1.1	5	21	26
% CI<1.1	86.7	13.3	100.0
CI<1.1	19.2	80.8	100.0

[1]83.9% of original grouped cases were correctly classified.

83.9% children had correct classifications, of which 86.7% was correctly classified as proportionate CI, and 5 (13.3%) were wrongly classified as disproportionate CI, 80.8% was correctly classified and 4 (19.2%) were wrongly classified.

-
