# Supplementary material for: Impact of Neonatal Body (Dis)Proportionality Determined by the Cephalization Index (CI) on Gross Motor Development in Children with Down Syndrome: A Prospective Cohort Study
Source: Children (Basel). 2022 Dec 21;10(1):13. doi: 10.3390/children10010013 (PMC9856915; doi:10.3390/children10010013)
Supplement: Supplementary file 1 [file children-10-00013-s001.zip › Supplementary Table S5_01.12.22.pdf]

Table S5. Casewise analysis of the division of children with Down syndrome (DS) into two groups based on the proportionality of anthropometric measures (cephalization index [CI]≥1.1 or proportionate [CI<1.1].

Casewise Statistics

	Case Number	Actual Group	Highest Group					Second Highest Group			Discriminant Scores
			Predicted Group	P(D>d | G=g)		P(G=g | D=d)	Squared Mahalanobis Distance to Centroid	Group	P(G=g | D=d)	Squared Mahalanobis Distance to Centroid	Function 1
				p	df						
Original	1	1	0**	,436	1	,972	,607	1	,028	7,207	-1,716
	2	0	0	,779	1	,850	,079	1	,150	3,033	-,716
	3	1	0**	,840	1	,868	,041	1	,132	3,281	-,790
	4	1	1	,228	1	,996	1,453	0	,004	12,895	2,410
	5	1	1	,853	1	,938	,034	0	,062	5,980	1,329
	6	1	1	,549	1	,978	,359	0	,022	8,468	1,767
	7	0	0	,839	1	,867	,041	1	,133	3,279	-,789
	8	1	1	,757	1	,814	,096	0	,186	3,567	,803
	9	0	0	,687	1	,950	,162	1	,050	5,522	-1,361
	10	1	1	,648	1	,968	,209	0	,032	7,563	1,616
	11	1	0**	,290	1	,563	1,117	1	,437	1,103	,017
	12	1	1	,437	1	,986	,605	0	,014	9,679	1,957
	13	0	0	,880	1	,878	,023	1	,122	3,448	-,838
	14	0	0	,549	1	,758	,359	1	,242	2,127	-,415
	15	1	1	,610	1	,972	,260	0	,028	7,897	1,673
	16	0	0	,831	1	,865	,046	1	,135	3,243	-,779
	17	0	0	,305	1	,981	1,054	1	,019	8,441	-1,950
	18	0	0	,330	1	,980	,951	1	,020	8,176	-1,902
	19	0	0	,349	1	,978	,877	1	,022	7,980	-1,865
	20	0	1**	,803	1	,835	,062	0	,165	3,827	,867
	21	0	0	,755	1	,843	,098	1	,157	2,934	-,686
	22	0	0	,569	1	,768	,325	1	,232	2,203	-,443
	23	1	1	,480	1	,983	,498	0	,017	9,181	1,881
	24	1	1	,847	1	,853	,037	0	,147	4,081	,927
	25	0	0	,679	1	,951	,172	1	,049	5,571	-1,372
	26	1	1	,581	1	,975	,305	0	,025	8,167	1,718
	27	0	0	,500	1	,967	,454	1	,033	6,715	-1,617
	28	0	1**	,546	1	,685	,364	0	,315	2,432	,492
	29	0	0	,226	1	,986	1,467	1	,014	9,422	-2,125
	30	1	1	,790	1	,949	,071	0	,051	6,432	1,414
	31	0	0	,888	1	,923	,020	1	,077	4,479	-1,114
	32	0	0	,670	1	,952	,182	1	,048	5,622	-1,384
	33	0	0	,453	1	,971	,563	1	,029	7,072	-1,689
	34	0	1**	,378	1	,532	,778	0	,468	1,553	,196
	35	1	1	,432	1	,587	,617	0	,413	1,835	,299
	36	0	0	,376	1	,644	,783	1	,356	1,449	-,145
	37	1	1	,759	1	,954	,094	0	,046	6,662	1,457
	38	1	0**	,545	1	,756	,367	1	,244	2,108	-,409
	39	0	0	,716	1	,946	,132	1	,054	5,359	-1,324
	40	1	1	,578	1	,975	,310	0	,025	8,193	1,722
	41	1	1	,999	1	,904	,000	0	,096	5,008	1,132
	42	0	0	,587	1	,777	,296	1	,223	2,272	-,467
	43	0	0	,862	1	,873	,030	1	,127	3,374	-,817
	44	1	1	,954	1	,916	,003	0	,084	5,300	1,193
	45	1	1	,500	1	,982	,455	0	,018	8,968	1,847
	46	0	0	,163	1	,989	1,948	1	,011	10,457	-2,299
	47	0	0	,283	1	,555	1,151	1	,445	1,074	,032
	48	1	1	,573	1	,976	,318	0	,024	8,239	1,730
	49	0	0	,105	1	,992	2,628	1	,008	11,796	-2,512
	50	0	1**	,907	1	,927	,014	0	,073	5,612	1,256
	51	1	1	,301	1	,993	1,070	0	,007	11,551	2,229
	52	1	1	,478	1	,629	,502	0	,371	2,076	,380
	53	1	1	,097	1	,999	2,753	0	,001	16,818	2,892
	54	0	0	,252	1	,984	1,314	1	,016	9,071	-2,063
	55	1	0**	,327	1	,599	,963	1	,401	1,250	-,054
	56	0	0	,515	1	,966	,423	1	,034	6,607	-1,595

**. Misclassified case

*Misclassified children. The discriminant analysis results (Supplementary Table 4) are based on casewise statistics. By analyzing the results in Supplementary Table 5, it was possible to determine which children with DS had a proportionate CI denoted as group 0 (without clinical characteristics) and a disproportionate CI denoted as group 1 (with clinical characteristics of disproportionality).
